# Beyond convention: non-compacted myocardium, ventricular tachycardia, and systolic dysfunction in dextrocardia patients: a case series

**DOI:** 10.1097/MS9.0000000000002855

**Published:** 2025-01-09

**Authors:** Natalia Nazarenko, Maisha Maliha, Luis Cerna, Nathaniel Abittan, Pawel Borkowski, Ibolya Csecs, Mario Garcia

**Affiliations:** a Department of Medicine, Jacobi Medical Center/AECOM, Bronx, New York; b Department of Medicine, Dhaka Medical College, Dhaka, Bangladesh; c Department of Cardiology, George Washington University, Washington, District of Columbia; d Department of Cardiology, Westchester Medical Center, Bronx, New York; e Department of Cardiology, Montefiore Medical Center, Bronx, New York

**Keywords:** case series, dextrocardia, genetic cardiomyopathy, heart failure with reduced ejection fraction (HFrEF), left ventricular noncompaction (LVNC), monomorphic ventricular tachycardia (VT)

## Abstract

**Background:**

Noncompaction of the left ventricle (LVNC) is linked to a higher risk of sudden cardiac death and stroke. Its prevalence ranges from 0.014% to 1.3% in the general population, rising to 7.5% in patients with dextrocardia.

**Case summary:**

A male in his late 70s presented with worsening dyspnea and leg swelling, with dextrocardia and frequent extrasystoles. Imaging revealed right-sided pleural effusion, severely reduced ejection fraction, and ventricular and atrial dilatation. He developed sustained monomorphic VT, was treated with amiodarone, had successful coronary stenting, and received an ICD with no further hospital readmissions or ICD events. A second case involved a male in his late 50s who presented with dyspnea. He had dextrocardia with situs inversus, subsegmental pulmonary embolism, and LVNC. He was treated with enoxaparin, medical therapy, and Holter monitoring, which showed mild arrhythmias. He declined ICD placement but remained event-free during the first year of follow-up.

**Discussion:**

LVNC is a rare condition resulting from abnormal myocardial development during embryogenesis, leading to a two-layered myocardial structure. Diagnosis is based on imaging criteria. LVNC is linked to arrhythmias, heart failure, and conduction abnormalities, requiring interventions such as ICD placement, arrhythmia monitoring, and genetic testing. Further research is needed on genetic associations and long-term outcomes in dextrocardia patients.

## Graphical abstract

**Figure:**
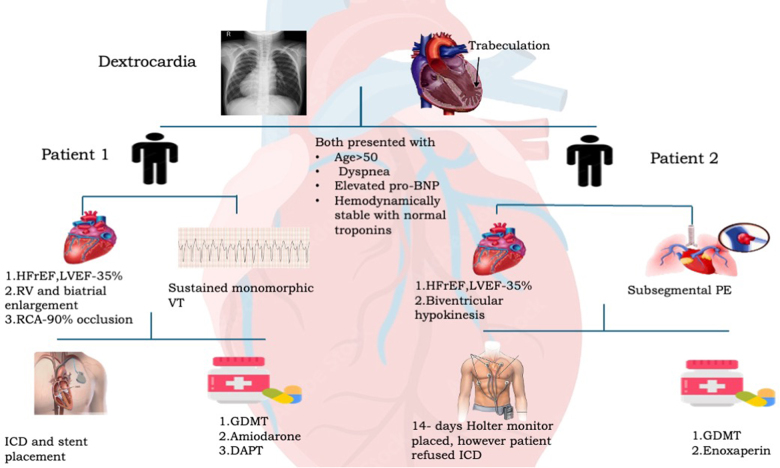

## Introduction

The incidence of left ventricular noncompaction (LVNC) among patients with dextrocardia surpasses that in individuals with a physiologically normal heart position by approximately 6%^[1]^. The etiology of LVNC is associated with a disrupted compaction process during embryogenesis, leading to the formation of deep intertrabecular recesses between the abnormal trabeculations^[2]^. LVNC is linked to a genetic predisposition in 40% of instances, more prevalent in men than in women, and marked by a range of clinical phenotypes, including congenital heart diseases due to various gene penetrations^[3]^. Among clinical manifestations, the most frequent include heart failure, thromboembolic events, and arrhythmias, particularly ventricular tachyarrhythmias^[4]^.

**Highlights**

- LVNC is more prevalent in individuals with dextrocardia, presenting significant risks such as heart failure and life-threatening arrhythmias. Awareness of this association can guide interventions like ICD placement and GDMT initiation.
- Diagnosis of LVNC relies on specific imaging criteria identifying abnormal trabeculations and non-compacted myocardium.
- LVNC has a genetic predisposition and may warrant genetic testing in patients with cardiomyopathy and dextrocardia.

The prevalence of LVNC is observed to be elevated in individuals with dextrocardia and situs inversus compared to those with a typical heart position. This case series aims to raise awareness within the medical community regarding the heightened likelihood of LVNC in patients with dextrocardia. Additionally, it aims to illustrate how LVNC can present as heart failure and, notably, as silent yet life-threatening ventricular tachycardia, necessitating fundamental changes in the treatment approach.

## Cases description

### Case #1

A male patient in his late 70s presented with worsening shortness of breath on exertion and lower extremity swelling lasting for 2 months. His medical history included dextrocardia and frequent extrasystoles. On physical exam, he had lung crackles, significant jugular venous distention, and 2+ pitting edema in his lower extremities. Vital signs were: blood pressure 94/81 mmHg, heart rate 53 bpm, respiratory rate 19, and oxygen saturation 95%. Blood tests showed an elevated B-type natriuretic peptide (BNP) at 1387 pg/mL (normal < 250). A chest X-ray revealed right-sided pleural effusion and confirmed dextrocardia.

**Figure 1.**
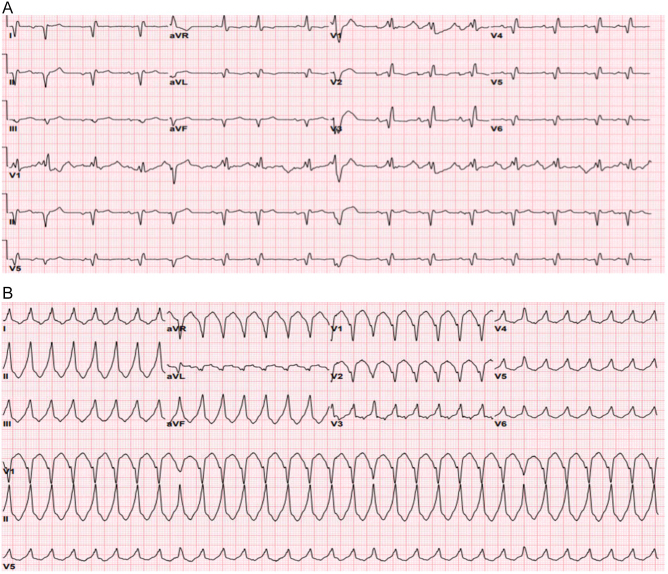
EKG. (1) Sinus rhythm with premature atrial and ventricular contractions. Extreme right axis deviation and poor R wave progression suggest dextrocardia. EKG. (2) Monomorphic sustained ventricular tachycardia. Left bundle branch block, inferior axis.

**Figure 2.**
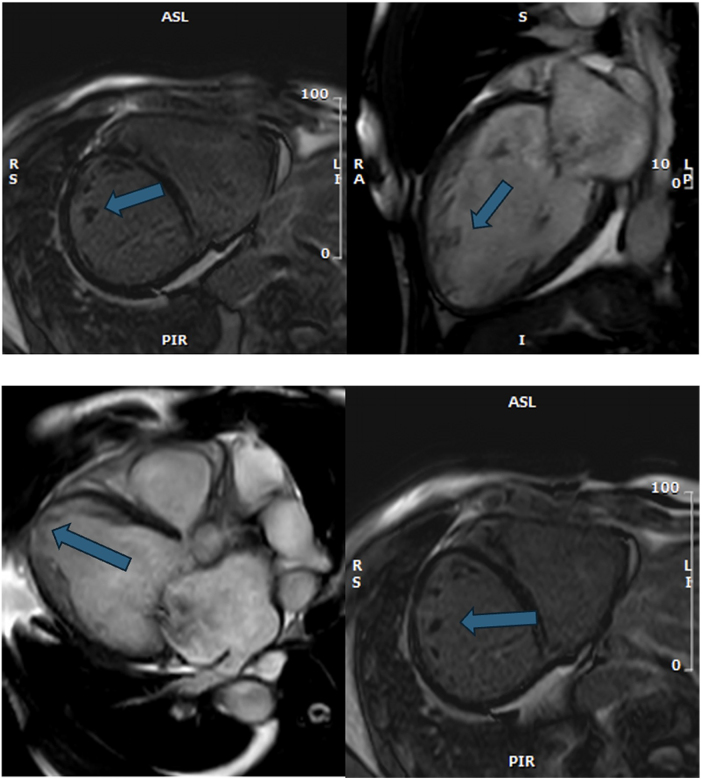
Cardiac MRI. Images (A, D) Hypertrabeculation in anatomical LV (short axis). Images (B, C) Hypertrabeculations are in the mid-apical part (sagittal localizer image).

Shortly after the initial evaluation, he developed palpitations and sustained monomorphic ventricular tachycardia (VT) with a heart rate of 180 bpm was observed (Fig. 1). He was treated with amiodarone and guideline-directed medical therapy (GDMT) for acute heart failure. An echocardiogram showed a severely reduced left ventricular ejection fraction (LVEF) of 35%, moderate left ventricular hypokinesis, moderate right ventricular enlargement, and severe dilation of both atria. Diagnostic heart catheterization revealed a 90% blockage in the middle right coronary artery, which was treated with a drug-eluting stent. A cardiac MRI confirmed dextrocardia and showed hyper-trabeculated left ventricular segments with severely reduced LVEF at 30% and right ventricular enlargement (Fig. 2).

Given the known arrhythmogenic risk, a dual-chamber implantable cardioverter defibrillator (ICD) was placed as secondary prevention. He was discharged on daily amiodarone, dual antiplatelet therapy, and GDMT for heart failure with reduced ejection fraction (HFrEF). Follow-up visits showed no ICD events or hospital readmissions.

### Case #2

**Figure 3.**
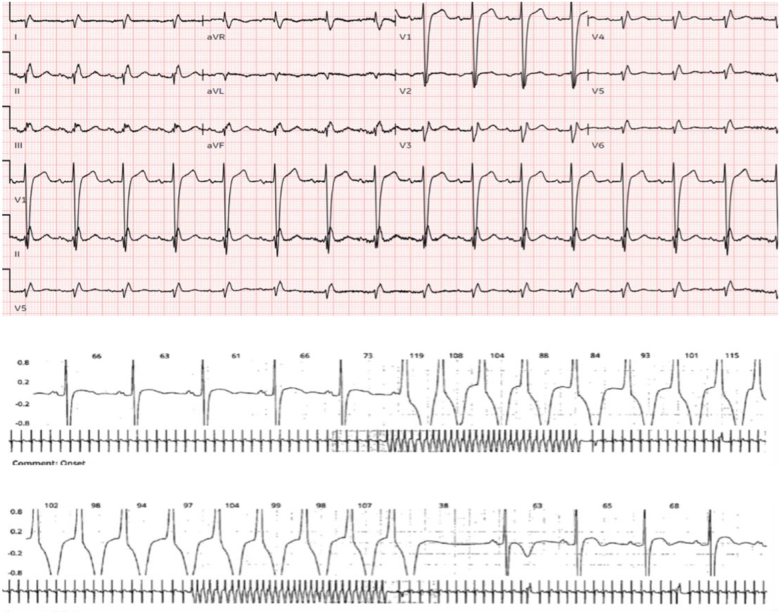
EKG. (1) Sinus rhythm, extreme right axis deviation with poor R waves progression in anterior leads, suggestive of dextrocardia. Holter. (2) Episode of non-sustained VT (31 beats) captured on Holter monitoring.

A male patient in his late 50s presented with 1 day of shortness of breath, chest tightness, and cough. He reported no past medical history and had not sought medical care for decades. Physical exam showed mild respiratory distress with significant bilateral wheezing. His vital signs were: heart rate 101 bpm, blood pressure 140/100 mmHg, and oxygen saturation 95%. Blood tests revealed normal troponin levels and pro-BNP at 151 pg/mL (normal 1–125). An ECG showed a regular sinus rhythm, extreme right axis deviation, and poor R-wave progression in the anterior leads (Fig. 3(1)).

**Figure 4.**
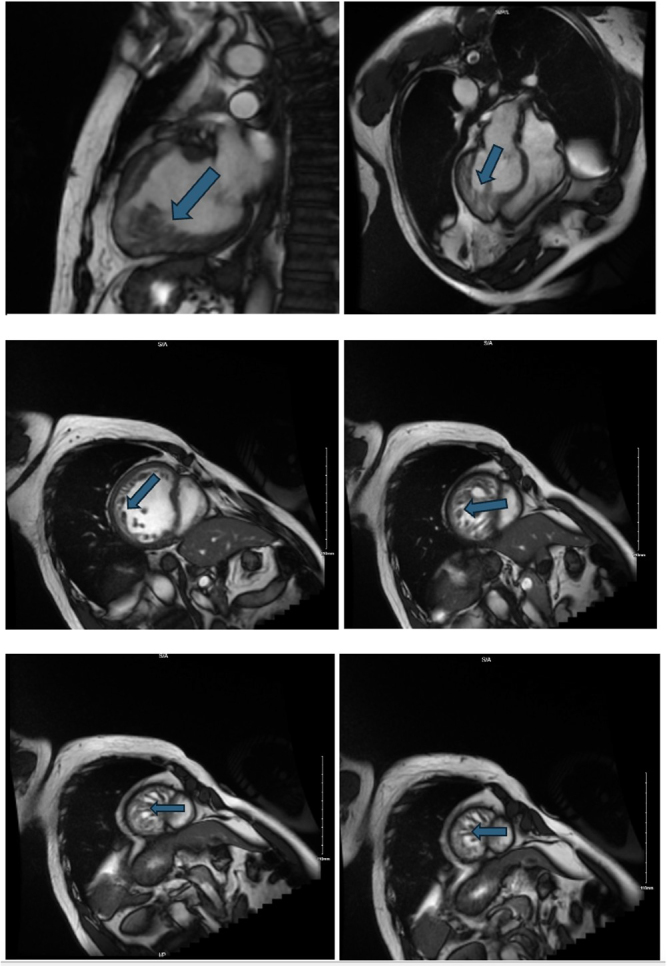
Cardiac MRI. Image (A) Hypertrabeculation in anatomical LV (sagittal localizer image). Images (B–F) Hypertrabeculation is more apparent in the mid-apical part (short axis bSSFP cine images).

A chest CT angiography was done to rule out pulmonary embolism, which showed dextrocardia with situs inversus, a subsegmental pulmonary embolism, and a markedly hypoplastic right pectoralis muscle. He was started on enoxaparin. An echocardiogram showed moderate left ventricular hypokinesis with an LVEF of 35% and increased trabeculations in the left ventricular apex. Heart catheterization showed no coronary artery disease. Cardiac MRI showed a dilated left ventricle with an LVEF of 35%, biventricular hypokinesis, and a non-compacted to compacted myocardium ratio of 5:1 without late gadolinium enhancement (Fig. 4).

GDMT was started, and he was discharged with 14-day Holter monitoring, which later showed two episodes of supraventricular tachycardia, 0.72% premature ventricular beats, and two episodes of non-sustained ventricular tachycardia (Fig. 3(2)). ICD placement was offered as secondary prevention, but the patient declined due to follow-up limitations. During the first year of follow-up, he had no hospital readmissions or cardiac events.

## Discussion

LVNC is a rare heart condition caused by developmental disturbances in the myocardium during early embryogenesis, leading to insufficient compaction of heart muscle fibers. First described by Grant in 1926, LVNC is characterized by a two-layered heart muscle structure: a thin compacted layer and prominent trabeculations with inter-trabecular recesses^[1,5]^. The American Heart Association (AHA) classifies LVNC as a genetic cardiomyopathy, while the European Society of Cardiology (ESC) and the World Health Organization (WHO) label it as a familial/genetic unclassified cardiomyopathy^[3]^. The estimated prevalence of LVNC in the general population is between 0.014% and 1.3%. However, Martinez *et al* studied 172 individuals with heterotaxy and found LVNC in 13 patients (7.5%), indicating a higher occurrence in heterotaxy patients^[5]^.

Specific diagnostic criteria for LVNC are still debated. In 2001, Jenni *et al*^[6]^ suggested transthoracic echocardiography criteria based on a non-compacted to compacted myocardium ratio > 2.0 in end-systole. Petersen *et al*^[7]^ proposed criteria for cardiac MRI (CMR), stating a non-compacted to compacted myocardium ratio > 2.3 in diastole, with 86% sensitivity and 99% specificity. Captur *et al* measured LV trabeculations using CMR and noted that patients with LVNC had a fractal dimension > 1.3, compared to 1.2 in healthy individuals, suggesting higher accuracy with this method^[8]^. In 2013, Stacey *et al* analyzed excessive trabeculations with a cutoff noncompaction-to-compaction ratio of ≥2.0 in 122 participants^[9]^.

LVNC subtypes include (a) benign LVNC (35% of cases), with normal LV function and no significant arrhythmias; (b) dilated LVNC, characterized by reduced LV function; (c) hypertrophic LVNC, with diastolic dysfunction and high systolic function; (d) hypertrophic-dilated LVNC, with higher mortality and metabolic issues, mainly in children; (e) restrictive LVNC, marked by atrial enlargement and diastolic dysfunction with arrhythmia risk; (f) Right ventricular or biventricular LVNC, with spongiform RV appearance; and (g) LVNC associated with congenital heart disease. LVNC can have various inheritance patterns, including autosomal recessive, autosomal dominant, X-linked, and mitochondrial^[2]^. Although most cases involve sarcomere- or cytoskeleton-related gene mutations, some, such as SCN5A, are linked to arrhythmias^[2]^. In heterotaxy syndrome, genes like ZIC3, LEFTYA, CRYPTIC, and ACVR2B are affected, although a link between these and LVNC has not been established^[10]^.

LVNC is associated with conduction issues, life-threatening arrhythmias, thromboembolic events, and heart failure^[11]^. A 20-year study involving 242 children found that 33.1% had arrhythmias, correlating with higher mortality (*P* = 0.002). Ventricular tachycardia (VT) occurred in 17.4% of cases, leading to resuscitated sudden cardiac death in some, and 6.2% experienced sudden cardiac death^[12]^. In a study of 960 patients, Kovacevic-Preradovic *et al* identified LVNC as the cause of heart failure in 2% of cases^[13]^. A systematic review of 135 LVNC cases with ventricular arrhythmias found that most originated in the non-compacted myocardium^[4]^. Recently, Peterson *et al* proposed changing the term LVNC to “excessive trabeculations,” suggesting that high trabeculations might not have independent prognostic value without clinical suspicion of an inherited condition, with risk more closely linked to low LVEF and myocardial injury^[14]^.

Decision-making in these cases was challenging due to the complex and overlapping clinical features of LVNC, dextrocardia, and situs inversus, which presented significant diagnostic and treatment hurdles. The lack of standardized diagnostic criteria for LVNC, along with evolving understanding of its clinical implications, made identifying and confirming the condition difficult. Furthermore, the patients’ structural abnormalities – such as severe ventricular dilation, arrhythmogenic substrate, and reduced ejection fraction – demanded precise assessment of arrhythmia risk and heart failure management strategies. The variability in clinical presentations, from heart failure symptoms to silent but life-threatening ventricular tachycardia, required nuanced judgment in implementing therapies like GDMT and determining whether invasive interventions, such as ICD placement, were warranted. Additionally, genetic considerations complicated decision-making, as the potential familial and heritable aspects of LVNC necessitated careful long-term planning, including family screening and preventive strategies. In the absence of prospective cohort data for such unique cases, clinicians had to balance immediate therapeutic needs with the potential long-term risks of heart failure and arrhythmia, highlighting the intricate nature of managing LVNC in patients with dextrocardia.

Our case series found an association between reduced LVEF and ventricular tachycardia in patients with LVNC and dextrocardia with situs inversus. This highlights the increased prevalence of noncompaction in individuals with dextrocardia and its possible link with ventricular arrhythmias and reduced LVEF. Awareness of these associations may prompt interventions such as genetic testing, arrhythmia monitoring, ICD placement, or GDMT initiation. Prospective data is needed to confirm these links and explore the genetics of cardiomyopathies and trabeculation disorders for better patient care.

Future criteria for studying and managing LVNC in patients with dextrocardia and situs inversus should prioritize integrating advanced imaging techniques like cardiac MRI to refine diagnostic accuracy, especially by addressing anatomical complexities. Emphasis should be placed on genetic testing to identify familial or heritable links, with a focus on sarcomere-related and heterotaxy-associated gene mutations. Clinical protocols could include stratifying patients based on LVEF and arrhythmia risk, guiding interventions like ICD placement or GDMT. Prospective data collection should explore how structural anomalies, such as ventricular dilation or excessive trabeculations, correlate with outcomes like heart failure and arrhythmias. Additionally, familial screening and long-term follow-up can provide insight into disease progression and inform preventive strategies.

The work has been reported as being in line with the PROCESS criteria.^[15]^

## Data Availability

Not applicable.
